# Antibacterial Effect of Eight Essential Oils against Bacteria Implicated in Bovine Mastitis and Characterization of Primary Action Mode of *Thymus capitatus* Essential Oil

**DOI:** 10.3390/antibiotics13030237

**Published:** 2024-03-05

**Authors:** Chedia Aouadhi, Ahlem Jouini, Karima Maaroufi, Abderrazak Maaroufi

**Affiliations:** 1 Laboratory of Epidemiology and Veterinary Microbiology, Group of Bacteriology and Biotechnology, Pasteur Institute of Tunisia (IPT), University of Tunis El Manar (UTM), BP 74, 13 Place Pasteur, Belvédère, Tunis 1002, Tunisia; ahlem.jouini@pasteur.tn (A.J.); abderrazak.maaroufi@pasteur.tn (A.M.); 2 Higher Institute of Biotechnology of Beja, University of Jendouba, Beja 9000, Tunisia; 3 Laboratory of Functional Physiology and Bio-Resources Valorization, Higher Institute of Biotechnology of Beja, University of Jendouba, Beja 9000, Tunisia; karimahedhli@yahoo.fr

**Keywords:** essential oil, antimicrobial activity, action mode, Staphylococci, Gram-negative bacteria, bovine mastitis

## Abstract

During the current investigation, eight essential oils (EOs) were tested for their antimicrobial activity against six species, belonging to the genus of *staphylococcus*, multi-resistant to antibiotics (*S. epidermidis*, *S. cohni*, *S. wareneri*, *S. scuiri*, *S. chromogenes*, *S. pasteuri*), three methicillin-resistant *Staphylococcus aureus* strains (MRSA) and two strains of *Escherichia coli,* producing extended-spectrum β-lactamase (ESBL) responsible for bovine mastitis. Our results indicated that the antimicrobial activities of eight EOs varied significantly among the types of EOs and bacterial species. *Thymus capitatus* and *Trachyspermum ammi* EOs display important antibacterial activity against all tested strains, with the inhibition zone diameters situated between 20 and 45 mm, while EOs of *Artemisia absinthium*, *Eucalyptus globulus*, *Eucalyptus camaldulensis*, *Myrtus communis* and *Mentha pulegium* exerted an intermediate activity. For *Cymbopogon citratus*, this effect depends on bacteria species. In fact, an important effect was observed against *S. warneri*, *S. epidermidis*, *S. cohenii*, *S. pasteuri* and MRSA (EC 39+) strains. In addition, the important lytic effect was observed against MRSA strains, showing that Gram-positive bacteria were more sensitive to *T. capitatus* EO than Gram-negative ones. Concerning the characterization of the mode action of *T. capitatus*, experiments of kill-time, bacteriolytic, loss of salt tolerance and loss of cytoplasmic material showed that the used EO was able to destroy cell walls and membranes followed by the loss of vital intracellular materials. In addition, it inhibits the normal synthesis of DNA, causing the bacterial death of *E. coli* and MRSA strains. This study shows the potential of using of EOs, particularly *T. capitaus,* to inhibit the growth of Gram-positive and Gram-negative bacteria multi-resistant to antibiotics causing bovine mastitis.

## 1. Introduction

Bovine mastitis is caused by several bacteria and, rarely, by yeasts, fungi, parasites or viruses [1]. This infection is often linked to trauma or attacks on the skin of the teat or at the level of the udder and sometimes to stenosis of the teat canal (lesions linked to the milking machine). The microorganisms causing mastitis are classified into three main categories, according to their sources, reservoir and their modes of transmission [1,2]. *Staphylococcus aureus* has been reported as the main causative agent of mastitis in cattle in several studies [3,4,5,6]. Furthermore, the emergence and spread of methicillin-resistant *Staphylococcus aureus* (MRSA) and methicillin-sensible *Staphylococcus aureus* (MSSA) are becoming a major concern for veterinary medicine as well as public health. MRSA is rarely detected as a causative agent of bovine mastitis in some countries, like Finland (1.5%), Germany (4.4%), Australia (4%) and Tunisia [6,7,8,9]. However, other studies reported the presence of MSSA: in Brazil (23%), Turkey (17.5%) and China (47.6%) [10,11,12]. This variability can be explained by the use of several categories of antibiotics belonging to the β-lactam family.

In addition to these contagious pathogens, *Enterobacteriaceae* are also the main environmental bacteria responsible for bovine mastitis. This family includes many genera, among which, we find *Escherichia coli*, which is frequently involved in mammary pathology [6,13,14]. In the same way, Klibi et al. [6] highlighted that the molecular characterization of Gram-negative bacteria and Staphylococci species showed a diversity of species with the respective dominance of *E. coli* (ten strains are extended-spectrum β-lactamase (ESBL) producers).

Generally, antibiotics are intended for the treatment of bovine mastitis. The presence of drug residues in milk constitutes a technological obstacle to the processing of milk and represents a danger to human health that is aggravated by the presence of multi-resistant bacteria. Since 2011, despite the World Health Organization (WHO) request to develop new drugs, a few molecules have been advanced. Plants can be considered as a source of natural bioactive substances that can be used in the manufacture of new antimicrobial drugs [15].

Recently, several findings have highlighted the different biological activities of essential oils (EOs), in particular their antibacterial power. In fact, Tariq et al. [16] signaled that EOs are more effective than conventional antibiotics against drug-resistant microbial strains. In a previous study, we investigated the antibacterial effect of eight EOs against *Escherichia coli* isolated from meat and able to produce extended-spectrum β-lactamase (ESBL) [17].

Although the chemical compositions and biological activities of EOs obtained from diverse plants were investigated, their antibacterial activities against Gram-negative and Gram-positive bacteria responsible for bovine mastitis have not been previously studied. In addition, until now, there are no published reports concerning the action mechanism of *T. capitatus* EO on the growth of *Staphylococcus* species multi-resistant to antibiotics. The aim of the current study is to evaluate the antibacterial effect of eight EOs (*Eucalyptus globulus*, *Eucalyptus camaldulensis*, *Artemisia absinthium*, *Myrtus communis*, *Mentha pulegium*, *Trachyspermum ammi*, *Cymbopogon citratus* and *Thymus capitatus)* against six species of *Staphylococcus* (*S. wareneri*, *S. epidermidis*, *S. scuiri*, *S. chromogenes*, *S. cohni*, *S. pasteuri*), methicillin-resistant *Staphylococcus aureus* (MRSA) and ESBLs producing *E. coli* strains. In addition, the characterization of the mechanism of antibacterial action of *T. capitatus* EO was investigated using four tests: kill-time analysis, bacteriolytic, loss of cytoplasmic material and loss of salt tolerance assays.

## 2. Materials and Methods

### 2.1. Plant Material

In the current report, eight essential oils (*Eucalyptus globulus*, *Eucalyptus camaldulensis, Artemisia absinthium*, *Myrtus communis*, *Mentha pulegium*, *Trachyspermum ammi*, *Cymbopogon citratus*, *Thymus capitatus)*, purchased from the company “Carthago Essences Sousse, Tunisia”, were used to study their antibacterial effect against different strains multi-resistant to antibiotics causing clinical bovine mastitis in Tunisia.

### 2.2. Selected Bacteria and Growth Conditions

In order to study the antimicrobial activity of eight EOs, six species of *Staphylococcus* (*S. wareneri*, *S. epidermidis*, *S. scuiri*, *S. chromogenes*, *S. cohni*, *S. Pasteursi*), three methicillin-resistant *Staphylococcus aureus* (MRSA) and two strains of *E. coli* (Ec CTX and Ec Mac), isolated and identified by Klibi et al. [6], were used. The determination of antibiotic susceptibility of different strains was carried out by Klibi et al. [18] according to the recommendations of the CLSI “Clinical Laboratory Standards Institute 2015” and the French Society of Microbiology. Table 1 summarizes the different characteristics of used strains.

These bacteria were maintained by sub-culturing on brain heart infusion agar (BHI agar), which was favorable to their growth for 24 h at 37 °C.

### 2.3. Screening of Antimicrobial Activity of EOs

The sensibility of different bacterial species to eight EOs was evaluated using the agar well diffusion method, as described by Aouadhi et al. [17]. The qualitative antimicrobial activity results of tested EOs are expressed as the diameter of the growth-inhibition zone (including the disc diameter of 6 mm).

### 2.4. Evaluation of the Quantitative Antibacterial Power of EOs

The determination of minimum inhibitory concentrations (MICs) and the minimum bactericidal concentrations (MBCs) was utilized to quantify the antibacterial effect of eights EO. In fact, broth dilution, described by Aouadhi et al. [19], was used. The working culture, obtained after incubation at 37 °C for 18 h, was performed in Mueller–Hinton broth from three to four colonies. Each tested EO was diluted from 0.07 to 50% (*v*/*v*) in dimethyl sulfoxide (DMSO).

The positive control corresponded to the bacteria without EO, and the negative control corresponded to the appropriate medium and test oil. After incubation for 24 h at 37 °C, the MICs and MBCs were determined. To confirm MICs and MBC, 100 µL of each tube was inoculated on BHI agar. After incubation at 37 °C, the count of survivor’s bacteria was determined. Each experiment was repeated at least three times.

### 2.5. Primary Mode of Action of T. capitaus EO

#### 2.5.1. Time-Kill Studies

This method makes it possible to characterize the antibacterial activity of *T. capitatus* EO over time against *E. coli* and MRSA strains. It evaluates the decrease in bacteria count in the presence of EO (at a concentration equal to its MIC) over several hours [20]. In fact, three to five bacterial colonies, obtained from an 18 h culture, are removed using a loop and deposited in 2 mL of peptone water. The obtained suspension was diluted to obtain a working culture (10^5^ CFU/mL). EO was used at a concentration equal to its MIC. The growth of bacterial suspensions in presence or in absence (growth control) of *T. capituatus* EO was monitored for 24 h at 37 °C under stirring. At different times (0, 2, 4, 6, 8 and 24 h), an aliquot of 100 µL was taken to estimate its rate [21].

#### 2.5.2. Bacteriolysis

This method allows for the determination of a possible bacteriolytic action of *T. capitatus* EO by measuring the absorbance of tested bacteria at 620 nm over time [21,22]. Indeed, two bacterial colonies, obtained from an 18 h culture, were used to inoculate 9 mL of nutrient medium. The culture was incubated at 37 °C for 18 h with shaking. The bacteria were then separated from the culture medium by centrifugation at 10,000 rpm for 12 min at 4 °C. The bacterial pellet was washed twice with sodium phosphate buffer (PBS), then resuspended in PBS-Tween 80 (0.01%, *v*/*v*). The obtained bacterial suspension was standardized at 10^10^ UFC/mL and placed in a sterile tube in the absence (negative control for bacteriolysis) or in the presence of EO at a concentration equal to the MIC. The obtained suspensions were subjected to stirring. After 120 min, they were homogenized, diluted to 1/100 and their absorbance was measured directly at 620 nm.

The results were expressed as a ratio (in percent) of the OD_620_ at each time point versus the OD_620_ at 0 min.

#### 2.5.3. Loss of Cytoplasmic Material

The release of 280 nm absorbing materials of two strains (*E. coli* and MRSA) in presence of *T. capitatus* EO was used to evaluate the loss of cytoplasmic material, according to previous method described by Carson et al. [22].

#### 2.5.4. Loss of Salt Tolerance

The loss of salt tolerance of *E. coli* and MRSA strains in the presence of *T. capitatus* EO was evaluated according to that previously described by Carson et al. [22]. In fact, untreated and treated suspensions of *E. coli* and MRSA strains, for 30 min with *T. capitatus* (the used concentration correspond to the MIC), were plated on nutrient agar containing different concentrations of NaCl (0 to 100 g/L). After incubation at 37 °C for 24 h, the colonies were counted. The numbers of UFC per milliliter on each nutrient agar plate were compared to those on the nutrient agar plate without NaCl.

### 2.6. Statistical Analysis

All experiments are expressed as the mean ± standard deviation (SD) of three replications. The obtained data were processed using Microsoft Excel 2007. Quantitative differences were assessed using the ANOVA procedure (SPSS 14.0 for Windows powershell 7.2) (*p* < 0.05) followed by Duncan’s multiple range test.

## 3. Results and Discussion

### 3.1. Antibacterial Effect of Essential Oils

During this study, the antimicrobial power of eight EOs against two strains of *E. coli* that is multi-resistant to antibiotics, producing extended-spectrum beta-lactamases (ESBL), six species belonging to the genus of *Staphylococcus* that are multi-resistant to antibiotics (*S. epidermidis*, *S. cohni*, *S. wareneri*, *S. scuiri*, *S. chromogenes*, *S. pasteuri*) and three MRSA strains causing bovine mastitis, was investigated.

The screening of qualitative and quantitative effects of different EOs was realized using three parameters: diameters of inhibition zones (IZs), MIC and MBC. The obtained data summarized in Table 2 and Table 3 clearly show that the antimicrobial activities of eight EOs varied significantly among the used EOs and bacteria species. According to Rossi et al. [23], the EOs are considered active if they produce microbial growth inhibition diameters equal to or greater than 20 mm. Considering this definition, we noted that *T. capitatus* and *T. ammi* EOs show important antibacterial activity against all tested strains, with IZ diameters situated between 20 and 45 mm, while the EOs of *A. absinthium*, *Eucalyptus*, *M. communis* and *M. pulegium* exerted intermediate activity. For *C. citratus*, this effect depends on bacteria species. In fact, an important effect was observed against *S. warneri*, *S. epidermidis*, *S. cohenii*, *S. pasteuri*, and MRSA EC 39+ strains. The lowest MIC (0.048% (*v*/*v*) was registered in the presence of *T. capitatus* and *T. ammi* EOs, and the highest MIC (12.5% (*v*/*v*) was observed in the presence of *M. pulegium* and *Eucalyptus* EOs. In addition, it can be signaled that the tested EOs were more active against *Staphylococcus* and MRSA strains than against *E. coli* strains. The diameters of the inhibition zone and the MIC varied between 14 and 45 mm and 0.048 and 12.5% for *Staphylococcus*, while, in the presence of *E. coli*, the diameters of the inhibition zone varied between 11 and 25 mm and the MIC situated between 0.39 and 6.25% for *E. coli*.

This difference in antimicrobial activity may be due to the difference in the chemical composition of the tested EOs. Generally, EOs are complex mixtures of natural compounds of about 20–60 constituents in varying quantities. The antimicrobial activity could be related to the major compounds of the EOs or to a synergistic effect between the major and minor compounds [24]. Nevertheless, some researchers reported that there is a relationship between the chemical composition and antimicrobial activity [25,26]. The highest antibacterial activity was observed in the presence of EO rich in aldehydes or phenols, such as carvacrol, eugenol or thymol, followed by EO containing terpene alcohol. EOs are inactive if the principal constituents were terpene hydrocarbons. However, weak activity was observed when the EOs containing ketone or esters such as β-myrcene, α-thujone or gerenyl acetate [27,28].

Thus, previous findings showed that the *T. capitaus* EO has very significant antibacterial activity compared to the other tested EOs, which might be due to its major constituents: thymol and carvacrol [17,29,30,31]. In previous studies, we demonstrated that thymol (81.49%), α-cubebene (3.44%), α-terpinene (3.83%) and β-ocimene (3.16%) were identified as the major components of *T. capitatus* [30]. In fact, Althunibat et al. [32] demonstrated that thymol and carvacrol exhibited important antimicrobial activity, with the most sensitive being the *P. aeruginosa* strain followed by *S. aureus*, *E. aerogenes* and *E. coli*. Moreover, EL Jalel et al. [26] signaled that *T. capitatus* from Abu-Draa has the highest antimicrobial activity against all tested strains compared to that from Sidi-Alhamery. Carvcrol was the major volatile component present in both essential oils of Abu-Draa and Sidi Al-Hamrey regions (58.56% and 24.28%, respectively) [26].

The antimicrobial activities of EO of *T. ammi* against *E. coli* and *Staphylococcus* species and MRSA are comparable to those obtained against *E. coli* strains isolated from meat. In fact, Aouadhi et al. [17] showed that EO extracted from *T. ammi* possesses important antibacterial activity, with the IZ situated between 20 and 22 mm, and the MIC ranged from 0.39 to 0.78% (*v*/*v*), respectively.

As shown in Table 2 and Table 3, it can be signaled that the EO of *M. pulegium* can be used as antimicrobial agent against pathogenic bacteria. These data are in agreement with those obtained by Aouadhi et al. [17], Ghazghazi et al. [33] and Ladjel et al. [34]. In addition, Amalich et al. [35] highlighted that the *M. pulegium* EO is more effective than amoxicillin against *E. coli*, *Pseudomonas aeruginosa* and *Klebsiella pneumoniae.* Concerning the chemical composition, Ghazghazi et al. [33] demonstrated that the main components of *M. pulegium* EO were menthone (41.7%), cis-isopulegone (31.71%) and isomenthone (15.03%).

Concerning *A. absinthium*, the antimicrobial activity of its EO depends on bacterial species. In fact, *S. warneri* and *S. epidermidis* were the most sensitive strains, with an MIC of 0.78% (*v*/*v*), while MRS strains were more resistant (MIC = 12.5%). In the same way, Belay et al. [36] demonstrated that EO obtained from *A. absinthium* is able to alter the biosynthesis of proteins, RNA, DNA and polysaccharide in the cells of *S. aureus,* causing the inhibition of its growth (MIC = 0.62 μL/mL). In addition, Riahi et al. [37] showed that EOs of *A. absinthium* harvested from four localities in Tunisia displayed antimicrobial activity (MIC varied between 12.5 and 25% (*v*/*v*)) against fungal strains, Gram-negative and Gram-positive bacteria. In addition, we demonstrated that the camphor, (Z)-sabinene hydrate and 1-terpinen-4-ol are the major compounds [37].

The antimicrobial activity of EO of *M. communis* observed in the current study may be due to alcoholic compounds such as borneol, having important antibacterial activity. In addition, other compounds belonging to ethers and hydrocarbon groups such as 1,8-cineole and α-pinene had moderate antimicrobial activity [38].

By analyzing the obtained data, we can conclude that, despite the antimicrobial effect of different EOs, it remains less important than that observed with the reference strains. This can be explained by the fact that the used bacteria have acquired different mechanisms of resistance to antibiotics, as shown in Table 1, and, therefore, have an impact on its resistance to different biological substances. In addition, the antimicrobial activity of used EOs does not depend on the structure of the bacterial cell wall and the arrangement of its outer membrane.

### 3.2. Characterization of Primary Action Mode of Thymus capitatus Essential Oil

#### 3.2.1. Dynamics of Action of EO by Measuring Bacterial Growth: Time-Kill Assay

**Figure 1:**
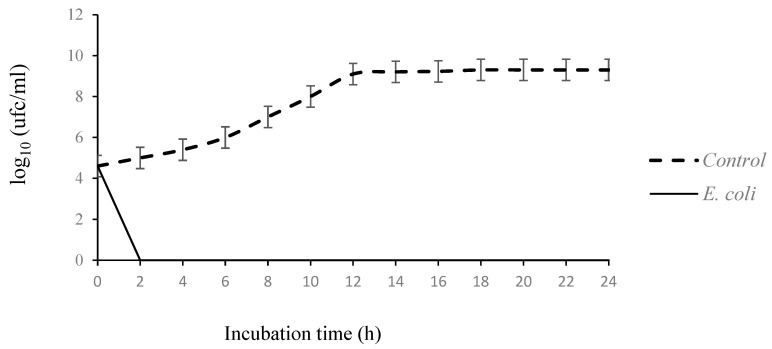

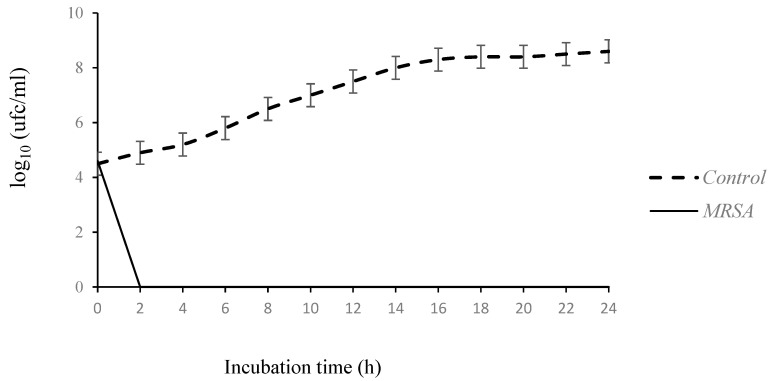
Time-kill curves *E. coli* and methicillin resistant *Staphylococcus aureus* cultures untreated and treated with the essential oil of *T. capitatus* at concentration corresponding to the minimum inhibitory concentrations. Values given are means (error bars represent standard deviations) of three independent experiments.

The bacterial growth of MRSA 44 and of *E. coli* strains, without or with (concentrations of essential oil equal to MIC) *T. capitatus* EO, was measured over a period of 24 h. MRSA and *E. coli* were selected as model microorganisms for further study based on their distinct sensitivities to *T. capitaus* EO. The choice of *T. capitatus* EO, for the study of the mode of action, was oriented to select the most active EOs against MRSA and *E. coli* based on the disk diffusion method and the determination of MIC and MBC values. As shown in Figure 1, the control case revealed a classic growth curve with three phases (growth, stationary and decline). In fact, the *E. coli* and MRSA 44 counts increased from 4.6 log_10_ to 9.3 log_10_ cfu/mL and 8.4 lg_10_ cfu/mL, respectively, after 24 h. In the presence of EO, the growth of two bacterial strains was inhibited after one hour, showing the important bactericidal effect of *T. capitaus* EO, supporting the results of MIC and MBC. In addition, the evaluated EO caused a decrease in the growth of *E. coli* within the first ten minutes. Our data corroborate a previous study about the rapid antibacterial effect of *T. capitatus* EO against *E. coli*–BLSE producers [17,29], probably because it contained a high concentration of carvacrol and thymol. Xu et al. [39] showed that carvacrol is able to embrittle and depolarize the cytoplasmic membrane. Moreover, after 30 min, the hydroxyl group of carvacrol and its isomers, thymol, causes an increase in their hydrophilic capacity, altering microbial cell formation [39].

#### 3.2.2. Bacteriolytic Effect

The screening for the bacteriolytic effect of *T. capitatus* EO against two species, MRSA and *E. coli,* is based on a measurement of the absorbance at 620 nm of bacterial suspensions in the absence or presence of EO, at a concentration equal to its MIC. The loss of absorbance is evaluated according to the initial absorbance. The obtained data clearly showed the absence of cell lysis of two used species in the absence of EO (the control case) since the absorbance was 100%. So, in the presence of EO, a decrease in the absorbance of two strains was observed. In fact, the percentage of OD decreased for *E. coli* to approximately 47% and for MRSA to 29%.

Based on this result, it can be concluded that *T. capitatus* EO exerts a bacteriolytic effect against *E. coli,* and MRSA species suggested that cell wall destruction is one of mechanism of action. The obtained data are comparable to those obtained by Aouadhi et al. [17], who demonstrated that *T. capitatus* exerts a bacteriolytic effect (OD in the presence of EO was 40%) against *E. coli,* producing extended-spectrum β-lactamases.

In addition, the important lytic effect was observed against MRSA compared to *E. coli,* showing that Gram-positive bacteria were more sensitive than Gram-negative ones towards the studied EO. This suggestion is consistent with previous studies carried out with several plant species [40,41] and is contrary to the investigation of Aouadhi et al. [30], who demonstrated that the EOs of *T. capitatus* harvested in two regions in Tunisia had powerful antimicrobial activity against all tested species (*B. cereus*, *L. monocytogens*, *S. aureus*, *E. coli*, *P. aeruginosa*, *A. hydrophila*, *S. typhimurium*).

Previous studies showed that the different antibacterial effects of some EOs against Gram-positive and Gram-negative bacteria can be attributed to the bacterial membrane. Indeed, the outer membrane of Gram-negative bacteria is rich in lipopolysaccharides. These molecules create a hydrophilic surface able to block the penetration of hydrophobic constituents of essential oils into the target cell membrane [40,41,42]. Consequently, *E. coli* producing extended-spectrum β-lactamases is relatively resistant to hydrophobic antibiotics. In contrast, for MRSA (Gram-positive bacteria), *T. capitatus* EO can block the enzyme system and progressivity of ion permeability thanks to the rupture of the cell membrane. Conner [43] highlighted that the thyme essential oil showed strong antibacterial activity against *Staphylococcus aureus*, *Salmonella typhi* and *Pseudomonas aeruginosa*. In addition, Horne et al. [44] showed that the EO of thyme generates a lytic effect.

#### 3.2.3. Loss of Salt Tolerance

The growth of *E. coli* and MRSA strains on nutrient agar containing different concentrations of NaCl, with or without *T. capiataus* EO, was used to study their salt tolerance. The obtained data summarized in Table 4 corroborated that the addition of NaCl in the medium, after exposure to *T. capitatus,* reduced the ability of bacteria to form colonies. In fact, in the absence of EO and the presence of different concentrations of NaCl (0 to 10%), the growth percentage of the two used strains was 100%, thus showing that the *E. coli* and MRSA tolerate a high concentration of NaCl, while in the presence of *T. capitatus* EO at a concentration equal to its MIC, the total absence of MRSA colonies at different concentrations and a very low percentage growth (1% at a concentration of 5% and 0.3% at a concentration of 10%) for *E. coli* strains were observed. This observation was in concordance with those obtained by Aouadhi et al. [17] when they studied the effect of two EOs (*T. capitatus* and *T. ammi*) against the salt tolerance of extended-spectrum β-lactamase (ESBL)-producing *E. coli* isolated from meat. The two used EOs significantly reduced the ability of bacteria to grow on media containing NaCl [17].

The obtained data highlighted clearly that the second mechanism of action of *T. capiatus* EO was the loss of osmoregulation of bacterial cells to salt, showing that the tested EO affected the envelope of the cell and cytoplasm. Similarly, Gilbert et al. [45] showed that the permeability and osmoregulation of the cell membrane can be affected by EO since the latter can lead to sublethal damage to the cell membrane of bacteria. Several authors explained that the EO can increase bacterial cell membrane permeability, causing a leak of cellular components and degradation of ions [22,46,47]. Also, it is able to reduce membrane potential and to disrupt proton pumps and the liquidation of ATP [48]. For example, Rudramurthy et al. [49] showed that carvacrol affects the membrane fluidity, and permeability damages the cell membrane by altering the composition of fatty acids.

#### 3.2.4. Loss of Cytoplasmic Material

Generally, the important macromolecules for cells are proteins and nucleic acids, which have an important role in cellular structure and genetic information [50]. The determination of absorbance at 260 nm (OD_260_) was used recently to understand the mechanism of action of EOs. This measurement is able to give us an idea of membrane integrity [51]. Table 5 summarizes the effect of *T. capitatus* EO on the integrity of the membrane of *E. coli* and MRSA. The OD_260_ of both filtrates after treatment increased significantly after 30 min, whereas in the control experiment, it remained practically stable (Table 5). Based on these data, it can be concluded that the EO of *T. capitatus* showed a release of absorbent constituents at 260 nm, so this oil affected the integrity and permeability of the membrane of *E. coli* and MRSA, leading to the leakage of nucleic acids through a damaged cytoplasmic membrane, inducing cell death. Bakkali et al. [52] showed that the EO damages mitochondrial membranes after it penetrates through the cytoplasmic membrane. Afterwards, free radicals, which oxidize and damage lipids, and proteins were produced by DNA in the mitochondria.

## 4. Conclusions

This is the first study to investigate the effect of different EOs against *E. coli* producing extended-spectrum β-lactamase (ESBL), six species belonging to the genus of *Staphylococcus* multi-resistant to antibiotics and three MRSA strains responsible for bovine mastitis. The obtained data highlighted the presence of a significant difference in the antibacterial activity between the eight EOs and according to the bacterial species. *T. capitatus* and *T. ammi* EOs showed important antibacterial activity against all tested strains, while the EOs of *A. absinthium*, *Eucalyptus*, *M. communis* and *M. pulegium* exerted intermediate activity. For *C. citratus*, this effect depends on bacteria species. Based on IZ, MIC and MBC values, it can be concluded that the used EOs possess antibacterial activity against Gram-positive and Gram-negative bacteria. But, using the cell lysis assay, Gram-positive bacteria are more sensitive to the action of essential oil than Gram-negative bacteria. *T. capitaus* EO (at a concentration equal to its MIC) affects the permeability and integrity of the cell membrane of *E. coli* and MRSA strains. In addition, it is able to cytoplasm material (DNA and proteins), causing cell death.

## Figures and Tables

**Table 1:** Characteristics of used bacterial strains [6,18].

Strains	Species	Phenotype of Resistance	Resistance Genes Detected
EC70BP−	*S. scuiri*	PEN, OXA, ERY, CLIN	*blaZ*, *mec*A, *erm*(B)
EC186BP+	*S. warneri*	PEN, OXA, FOX, CLIN, CIP	*blaZ*, *mec*A
EC196BP+	*S. epidermidis*	PEN, OXA, FOX, TET, SXT	*blaZ*, *mec*A, *Dfr*(A)
EC53+O	*S. pasteuri*	PEN, OXA, FOX, STR	*blaZ*, *mec*A
EC39BP−	*S. chromogenes*	OXA	*mec*A
EC60+O	*S. cohnii*	PEN, OXA, FOX, CLIN	*blaZ*, *mec*A
EC44BP+	Methicillin resistant *Staphylococcus aureus* (MRSA)	PEN, OXA, STR, ERY	*bla*Z, *Mec*A, *msr*A
EC159BP+		PEN, STR, OXA	*bla*Z, *Mec*A
EC39+O		PEN, OXA	*bla*Z, *Mec*A
172MAC	*E. coli*	TET, AMP, SXT, TIC, SUL, SMN	*TEM-1b*, *tetB*
78CTX		TET, AMP, SXT, TIC, SUL, SMN, CHL	*TEM-1b*, *tetA*, *catA*, *strB*

PEN: Penicillin, OXA: Oxacillin, ERY: Erythromycin, CLIN: Clindamycin, CIP: Ciprofloxacin, FOX: Céfoxitine, AMP: ampicillin; TIC: ticarcillin; TET: tetracyclin; SXT: sulfamethoxazole-trimethoprim; SUL: sulfonamide; SMN: streptomycin; CHL: chloramphenicol.

**Table 2:** Zone of inhibition expressed in diameter (mm) of eight essentials oils against extended-spectrum β-lactamase (ESBL)-producing *Escherichia coli*, six *staphylococcus* species and three MRSA strains.

Strains	Diameters of Inhibition Zone (mm)							
	*E. globulus*	*E. camaldulensis*	*A. absinthium*	*M. communis*	*M. pulegium*	*T. ammi*	*C. citratus*	*T. capitatus*
*S. scuiri*	13 ± 2	15 ± 2	12 ± 2	16 ± 2	13 ± 1.6	32 ± 1	12 ± 1	35 ± 1
*S. warneri*	14 ± 0.5	20 ± 1	16 ± 1	19 ± 1	17 ± 1	33 ± 5	40 ± 0.66	40 ± 2
*S. epidermidis*	28 ± 0.5	18 ± 0.5	19 ± 1	18 ± 2	14 ± 2	30 ± 2	40 ± 0.33	28 ± 0.5
*S. pasteuri*	12 ± 0.5	16 ± 1	20 ± 0.5	23 ± 1	13 ± 1	36 ± 2	26 ± 1.2	30 ± 1
*S. chromogenes*	16 ± 0.5	11 ± 0.5	14 ± 2	15 ± 2	15 ± 2	33 ± 2	15 ± 1.3	33 ± 3
*S. cohnii*	18 ± 1	15 ± 2	16 ± 1	13 ± 1	12±0.5	28 ± 1	30 ± 2	24±1
MRSA EC44BP+	14 ± 0.9	16 ± 2	14±1	21 ± 1	15 ± 0.5	40 ± 1.5	32 ± 2	45 ± 2
MRSA EC159BP+	15 ± 0.6	16 ± 1	17 ± 3	16 ± 0.5	15 ± 1	25 ± 0.5	18 ± 1.5	26 ± 2
MRSA EC39+O	14 ± 0.5	19 ± 0.5	15 ± 2	16 ± 2	14 ± 0.5	34 ± 2	44 ± 0.5	34±0.4
*E. coli* 172MAC	12 ± 1.5	13 ± 1	11 ± 1	11 ± 1	14 ± 0.5	22 ± 1	13 ± 1	25 ± 0.5
*E. coli* 79CTX	13 ± 0.5	18 ± 1	12 ± 1	20 ± 2	11 ± 1	20 ± 1.5	11 ± 0.5	25 ± 1.5

**Table 3:** Minimum inhibitory concentrations and minimum bactericidal concentrations (% *v*/*v*) of the tested essentials oils.

**Strains**	**Minimum Inhibitory Concentrations (% *v*/*v*)**							
	***E. globulus***	***E. camaldulensis***	***A. absinthium***	***M. communis***	***M. pulegium***	***T. capitatus***	***T. ammi***	***C. citratus***
*S. scuiri*	12.5	1.56	12.5	3.12	12.5	0.39	0.39	6.25
*S. warneri*	1.56	1.56	0.78	3.125	6.25	0.097	0.097	3.125
*S. epidermidis*	12.5	1.56	0.78	6.25	12.5	0.195	0.195	6.25
*S. pasteuri*	12.5	1.56	6.25	0.78	12.5	0.097	0.097	6.25
*S. chromogenes*	3.125	0.78	3.125	25	3.12	0.048	0.048	6.25
*S. cohnii*	3.125	1.56	6.25	6.25	12.5	0.048	0.048	0.78
MRSA EC44BP+	6.25	12.5	12.5	3.125	6.25	0.048	0.048	3.125
MRSA EC159BP+	6.25	12.5	3.125	3.125	12.5	0.195	0.195	3.125
MRSA EC39+O	6.25	3.125	12.5	3.125	12.5	0.048	0.048	3.125
*E. coli* 172MAC	1.56	0.78	3.12	6.25	3.12	0.39	0.39	3.12
*E. coli* 79CTX	6.25	6.25	3.12	1.56	3.12	0.39	0.39	1.56
**Strains**	**Minimum Bactericidal Concentrations (% *v*/*v*)**							
	***E. globulus***	***E. camaldulensis***	***A. absinthium***	***M. communis***	***M. pulegium***	***T. capitatus***	***T. ammi***	***C. citratus***
*S. scuiri*	25	3.125	25	6.25	25	0.78	1.56	12.5
*S. warneri*	3.125	3.125	1.56	3.125	12.5	0.197	0.39	6.25
*S. epidermidis*	25	3.125	1.56	6.25	25	0.39	1.56	12.5
*S. pasteuri*	25	3.125	12.5	6.25	25	0.195	0.39	12.5
*S. chromogenes*	6.25	1.56	6.25	6.25	6.26	0.097	0.78	12.5
*S. cohnii*	6.25	3.125	12.5	0.78	25	0.097	0.39	1.56
MRSA EC44BP+	12.5	25	25	3.125	12.5	0.097	0.78	6.25
MRSA EC159BP+	12.5	25	6.25	3.125	25	0.39	0.097	6.25
MRSA EC39+O	12.5	6.25	25	3.125	25	0.097	0.78	6.25
*E. coli* 172MAC	3.12	1.56	6.25	12.5	6.25	0.78	0.78	6.25
*E. coli* 79CTX	12.5	12.5	6.25	3.12	6.25	0.78	0.78	3.12

**Table 4:** Effect of various concentrations of NaCl on the growth of *E. coli* and MRSA in the presence of *T. capitatus* EO at a concentration equal to its minimum inhibitory concentrations.

Strains	Percentage of Strain Growth (%)					
	Control			*T. capitatus*		
	2.5%	5%	10%	2.5%	5%	10%
**MRSA**	100	100	100	0	0	0
***E. coli***	100	100	100	0	1	0.3

**Table 5:** Effect of *T. capitatus* EO on the absorbing at OD_620_ material of *E. coli* and MRSA strains.

Strains	Percentage of Initial OD_620_					
	Control			*T. capitatus*		
	0	30 min	60 min	0	30 min	60 min
**MRSA**	1	1.06	1.12	1	2.87	2.99
***E. coli***	1	1.02	1.13	1	2.3	2.5

## Data Availability

Data included in article/referenced in article.
